# Quantum Well-Enhanced Plasmonic Substrate to Enhance Spontaneously Blinking Fluorescence for Single-Molecule Localization Microscopy

**DOI:** 10.1021/acs.analchem.5c03721

**Published:** 2026-03-10

**Authors:** Shang-En Hsieh, Jian-Zong Lai, Kun-Yu Lai, Wan-Chen Huang, Fan-Ching Chien

**Affiliations:** † Department of Optics and Photonics, 34911 National Central University, Taoyuan 32001, Taiwan; ‡ Single-Molecule Biology Core Lab, Institute of Cellular and Organismic Biology, Academia Sinica, Taipei 115201, Taiwan; § Institute of Medical Device and Imaging, National Taiwan University, Taipei 10051, Taiwan

## Abstract

Quantum well (QW)-enhanced plasmonic substrates have been demonstrated to improve the blinking fluorescence of spontaneously blinking fluorophores, which enhances the localization precision and density for single-molecule localization microscopy (SMLM). The QW-enhanced plasmonic substrate consists of a three-repeat InGaN QW structure covered by Al nanoparticles. In addition to the localized surface plasmon enhancement produced by Al nanoparticles, InGaN QWs with tunable discrete energy levels and a high-density surface charge distribution can facilitate additional charge transfer resonances. This effect further enhances the local surface plasmon resonance around the Al nanoparticles. Moreover, the interaction between the high-density surface charges of the InGaN QWs and the oscillating electrons of the Al nanoparticles can lead to another type of surface plasmon enhancement effect. Therefore, the blinking intensity and event frequency are significantly increased, resulting in improved SMLM image resolution under the wide-field fluorescence excitation. With multiple fluorescence enhancement effects, the QW-enhanced plasmonic substrate enables SMLM imaging of phosphorylated epidermal growth factor receptors (EGFRs) in A549 lung cancer cells to quantitatively investigate the inhibition of EGFR tyrosine kinase. Furthermore, this QW-enhanced plasmonic substrate can reduce the excitation power needed for SMLM imaging at an acceptable resolution.

## Graphical abstract

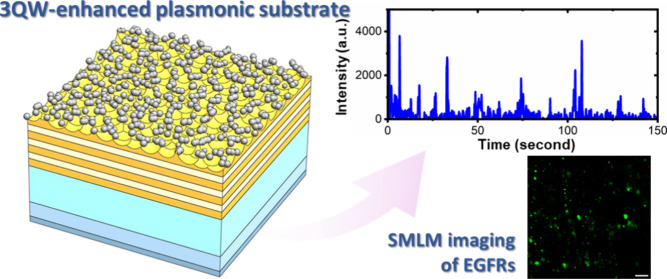

Single-molecule localization microscopy (SMLM) utilizes fluorescence blinking to obtain a sparse distribution of diffraction-limited fluorescent spots on fluorophore-labeled specimens.^1–4^ Through single-molecule localization and reconstruction analysis, a super-resolution fluorescence image can then be achieved. The blinking signals of labeled fluorophores can be generated through mechanisms such as photoactivation, photoconversion, photoswitching, and spontaneous blinking.^1,4–7^ Single-molecule localization imaging using spontaneously blinking fluorophores offers many advantages, such as single-wavelength excitation, low excitation power density, long-term live-cell imaging capability, and no need for additional special imaging reagents.^1,8–10^ Rhodamine derivatives and xanthene-based fluorophores have been synthesized to generate spontaneously blinking fluorescence signals for single-molecule localization imaging.^7,11–14^ Rhodamine derivatives and xanthene-based fluorophores achieve the fluorescence blinking phenomenon through the intramolecular spirocyclization reactions and the nucleophilic addition of intracellular glutathione, respectively. However, spontaneously blinking fluorophores typically produce fewer detectable photons per localization event than commercially available dyes used for direct stochastic optical reconstruction microscopy.^15^ Consequently, improving the number of detectable photons per localization event from spontaneously blinking fluorophores could significantly enhance the localization precision and reduce the excitation power density requirements in SMLM imaging.

The surface plasmon effect induced on metal nanoparticles and nanostructures can be utilized to manipulate their local density of states.^16–19^ This modulation influences the photophysical properties of nearby fluorophores, affecting aspects such as the excitation rate, the radiative decay rate, and the fluorescence lifetime. By increasing the excitation and radiative decay rates of a fluorophore while decreasing its fluorescence lifetime, a higher number of emitted photons can be obtained from a single fluorophore and less photobleaching effect can be obtained, significantly improving stability in fluorescence.^20–22^ Plasmon-enhanced fluorescence from gold nanorods uses the strong spectral overlap between the longitudinal plasmon resonance of the nanorods and the emission from the fluorophore, leading to enhanced single-molecule fluorescence.^23^ This enables the detection of individual crystal violet molecules on the nanorod surface even under a high-concentration environment. Moreover, the local surface plasmon resonance of gold nanorods also enhances both the excitation and radiative decay rates of photoactivatable fluorescent proteins, thus improving their brightness and photostability.^24^ As a result, the total number of photons emitted from a photoactivatable fluorescent protein during the photoactivated localization microscopy imaging can increase by up to 1.4 times. Furthermore, using the single-molecule localization technique, the spatial distribution of optical near fields and the local density of states on plasmon-enhanced nanostructures can be resolved at super-resolution scales.^18,25^ Additionally, surface plasmon-coupled emission substrates are also used to enhance single-molecule blinking fluorescence signals to reduce the excitation power density required for SMLM imaging.^15^ The uniform distribution in optical resonance coupling provided by such substrates contributes to revealing the real spatial distribution of fluorophore-labeled molecules in SMLM imaging. The Purcell effect provided by metamaterial substrates enhances the photoluminescence intensity, blinking rate, and photostability of dye molecules, ultimately improving the localization precision.^26^

The introduction of a charge transfer mechanism presents significant potential as an alternative method to enhance the fluorescence signals of fluorophores. The charge transfer effects from nonradiative decay, excited-state electron transfer, and energy transfer within fluorophores typically result in fluorescence quenching.^27–29^ However, research has shown that optimal energy band alignment between polypyrrole nanowires and silver nanoparticles can effectively enhance resonant charge transfer, leading to improved fluorescence and surface-enhanced Raman scattering (SERS) signals.^30^ Furthermore, when light excites a mixed thin film composed of the organic p-type conducting polymer poly­(3-hexylthiophene-2,5-diyl) and silver nanoparticles, charges are transferred to the silver nanoparticles.^31^ This leads to an enhanced electromagnetic field and an increased fluorescence intensity of the rhodamine B molecule. Our previous work also reported that alignment of energy bands at the interface of rhodamine 6G (R6G) molecules, metal, and InGaN quantum wells (QWs) can facilitate charge transfer and localized surface plasmon resonance within the R6G/metal/QWs layer structure.^32^ This interaction enhances the SERS signals of the R6G and DNA molecules.^32,33^ Moreover, by controlling the number of QW layers, the charges can be highly concentrated and increased at the surface of the substrate, further enhancing both the intensity and the density of hotspots on the substrate. Therefore, we presented a multilayer semiconductor QWs substrate decorated with metal nanoparticles to enhance the fluorescence signals of spontaneously blinking fluorophores for SMLM imaging. The three repeated composite layers of InGaN/GaN on a sapphire substrate were fabricated to form InGaN QWs structures. A layer of aluminum (Al) nanoparticles was covered on the InGaN QWs surface to obtain the 3QW-enhanced plasmonic substrate. The structure composed of fluorophores, Al nanoparticles, and InGaN QWs can enhance fluorescence through multiple mechanisms. These include additional channels for charge transfer resonance, a high concentration of surface charges, local surface plasmon resonance from Al nanoparticles, and surface plasmon coupling between Al nanoparticles and InGaN QWs.^34–36^ Consequently, the blinking fluorescence and blinking events of spontaneously blinking fluorophores can be significantly enhanced on 3QW-enhanced plasmonic substrates, thereby improving localization precision and image resolution in SMLM imaging. This SMLM image of phosphorylated epidermal growth factor receptors (pEGFRs) in A549 lung cancer cells on 3QW-enhanced plasmonic substrates can further be used to investigate inhibition of EGFR signaling under treatment with tyrosine kinase inhibitor (TKI).

## Experimental Section

### Materials and Reagents

The HM Janelia Fluor 526 (HM-JF_526_) NHS ester was purchased from Tocris Bioscience. AffiniPure goat antimouse IgG (H + L) was purchased from Jackson ImmunoResearch Laboratories, Inc. The sodium bicarbonate, dimethyl sulfoxide, paraformaldehyde, and fibronectin were obtained from Sigma-Aldrich. Dulbecco’s modified Eagle’s medium (DMEM) and fetal bovine serum (FBS) were purchased from Cytiva. A549 lung cancer cells were obtained from the Bioresource Collection and Research Centre, Taiwan. Monoclonal anti-EGFR antibody (A-10) and monoclonal p-EGFR antibody (SC57545) were purchased from Santa Cruz Biotechnology, Inc. Gefitinib (HY-50895) was ordered from MedChemExpress (USA).

### Fabrication of 3QW-Enhanced Plasmonic Substrates

The metal–organic chemical vapor deposition (MOCVD) system (AIXTRON 200/4 RF) was used to fabricate the three-repeat quantum wells on the c-plane sapphire substrate, as previously reported.^33^ In the MOCVD process, the carrier gases for n-type GaN and InGaN QWs were hydrogen and nitrogen, and the precursor materials were ammonia, trimethylgallium, triethylgallium, and trimethylindium. MOCVD pressure was set at 200 mbar. First, a nucleation layer of GaN approximately 25 nm thick was grown as a buffer layer to reduce defects caused by lattice mismatch. This was followed by the deposition of a 2 μm-thick n-type GaN layer, which serves as a high quality GaN base for the subsequent growth of InGaN quantum wells. Next, a three-repeat InGaN quantum well structure was fabricated by sequentially depositing 3 nm thick n-type GaN and 2 nm thick InGaN quantum wells, repeated three times. A 1.6 nm thick GaN cap layer was finally deposited to protect the InGaN QW structure. The deposition temperatures and thicknesses of each layer on the three-repeat QW (3QW) substrate are listed in Table S1. After sequentially washing with acetone, isopropyl alcohol, and deionized water, the 3QW substrate was coated with a 30 nm thick Al film using an e-beam evaporator (ULVAC). Finally, the substrate was rapidly thermally annealed at 300 °C for 3 min, resulting in the decoration of Al nanoparticles on the surface of the 3QW-enhanced plasmonic substrate. Additionally, we also fabricated a substrate without the three-repeat InGaN quantum well structure (i.e., a 0QW-enhanced plasmonic substrate) for comparison.

### Preparation of Blinking Fluorescence Specimens

To investigate the blinking fluorescence characteristics of single fluorophores, we followed a procedure similar to that described in previous reports to prepare spontaneously blinking fluorophores on the three substrates.^37,38^ The sapphire substrate, 0QW-, and 3QW-enhanced plasmonic substrates were immersed in a 1× phosphate-buffered saline (PBS) solution containing 2 μM fibronectin for 12 h. After being rinsed three times with PBS, a layer of fibronectin was attached to the substrate surface. Fibronectin is one of the extracellular matrix proteins. This layer of extracellular matrix proteins promotes cell adhesion and creates a spacing between the fluorophore and the Al nanoparticle, preventing fluorescence quenching from their close proximity. The three types of substrates were immersed in a PBS solution containing 1 nM Hydroxymethyl Janelia Fluor 526 (HM-JF_526_) for 4 h. After being rinsed three times with PBS, the three HM-JF_526_-coated substrates were placed in a chamber containing PBS solution and covered with a glass coverslip. Subsequently, the blinking fluorescence signals of a single HM-JF_526_ fluorophore were measured using a fluorescence imaging system. Furthermore, a filtering procedure was implemented to remove fluorescence blinking events that may have resulted from fluorophore aggregation. For each identified blinking event, we tracked the centroid position of the fluorescence-on signal on a frame-by-frame basis. Fluorescence-on signals exhibiting a centroid displacement greater than 20 nm between consecutive frames were regarded as originating from two or more distinct fluorophores in close proximity and were excluded from the statistical analysis. This approach could effectively remove data associated with fluorophore aggregation from the final analysis.

### Preparation of HM-JF_526_-Labeled Lung Cancer Cell Specimens

To immunostain the EGFR and pEGFR of A549 lung cancer cells, HM-JF_526_ fluorophores were conjugated to antimouse IgG as secondary antibodies. According to the manufacturer’s protocol, antimouse IgG was exchanged buffered in 0.1 M NaHCO_3_ (pH 8.4) solution using an ultrafiltration filter (Vivaspin 500; Sartorius). HM-JF_526_ NHS ester in a dimethyl sulfoxide solution was added to the antimouse IgG solution at a 10-fold molar excess of IgG proteins. The mixture of HM-JF_526_ NHS ester and antimouse IgG was incubated overnight at 4 °C in the dark. The unconjugated HM-JF_526_ NHS ester and antimouse IgG were removed by a PD SpinTrap G-25 column (GE HealthCare) to obtain secondary antibodies conjugated with HM-JF_526_ for immunostaining.

A549 lung cancer cells were maintained in Dulbecco’s modified Eagle medium (DMEM) supplemented with 10% (v/v) fetal bovine serum, 2 mM l-glutamine, and 1.5 g/L NaHCO_3_ at 37 °C in a humidified atmosphere containing 5% CO_2_. The glass coverslip, 0QW-, and 3QW-enhanced plasmonic substrates were coated with a layer of fibronectin as previously described in the above section. A total of 5 × 10^4^ A549 lung cancer cells were seeded onto the three substrates for 24 h of incubation. For EGFR immunostaining, A549 lung cancer cells were fixed with 4% paraformaldehyde in PBS for 20 min and permeated with 0.1% Triton X-100 for 15 min. The cells were then immersed in a blocking solution of 1% bovine serum albumin in PBS overnight at 4 °C. After washing cells three times with PBS, cells on the three substrates were incubated overnight at 4 °C with a primary monoclonal anti-EGFR antibody and a primary antitubulin antibody at a concentration of 5 μg/mL. Subsequently, a secondary antibody conjugated with HM-JF_526_ was incubated for 4 h at room temperature at a concentration of 2 μg/mL. After three washes with PBS, HM-JF_526_-conjugated EGFRs and tubulins in A549 lung cancer cells were used to acquire SMLM images.

To investigate the variation in pEGFR levels of A549 lung cancer cells treated with Gefitinib, 5 × 10^4^ A549 lung cancer cells were incubated on 3QW-enhanced plasmonic substrates for 24 h. Subsequently, the culture medium was replaced with a fresh medium containing Gefitinib at different concentrations (0, 5, and 10 μM) for an additional 12 h of incubation. After washing the samples three times with PBS, we followed the same immunostaining protocol described above to label the pEGFRs in A549 lung cancer cells. This was achieved using a 10 μL/mL primary monoclonal anti-pEGFR antibody and 2 μL/mL of a secondary antibody conjugated to HM-JF_526_.

### Measurement of Photoluminescence and Fluorescence Spectra

The photoluminescence spectra of the 3QW-enhanced plasmonic substrates were measured using a single longitudinal mode laser with a wavelength of 488 nm, which was focused through an objective lens (100× LMPlanFl, Olympus) for excitation. The emitted photoluminescence was collected through the same objective lens, then filtered using a dichroic mirror (Semrock) and an edge filter (Semrock), and recorded with a spectrometer (Shamrock SR-303i, Andor) coupled to an electron-multiplying charge-coupled device (EMCCD) camera (iXon Ultra 897, Andor). The excitation power and integration time were set at 3.6 mW and 50 ms, respectively. The fluorescence signals of R6G on sapphire, 0QW-, and 3QW-enhanced plasmonic substrates were also compared using this spectral measurement system. After coating with a layer of fibronectin as described above, each substrate was immersed in a 1 μM R6G solution for 4 h. The three substrates were rinsed three times with ethanol and dried with nitrogen gas, and the R6G fluorescence spectra were measured on each substrate to evaluate their fluorescence enhancement. Additionally, absorption spectra of 0QW- and 3QW-enhanced plasmonic substrates were measured using a HITACHI U4100 spectrophotometer. Absorption was calculated by subtracting the sum of reflectance and transmittance from the total incident intensity at a 10° incident angle.^39^

### Numerical Simulation of Electrical Field Distribution

The electromagnetic field distribution of the 3QW-enhanced plasmonic substrate was calculated using a two-dimensional finite-difference time-domain method. The thicknesses of the individual layers comprising the 3QW structure are summarized in Table S1, and the dielectric constants of each layer were adopted from previously published values.^33^ In the simulation, an Al nanoparticle layer was placed on top of the 3QW substrate. A single AlO_*x*_ layer was assumed to form on the Al nanoparticle surface, and a fibronectin layer was subsequently added above the AlO_*x*_. The thicknesses of the AlO_*x*_ and fibronectin layers were set to 5 and 4 nm, respectively.^33,40^ The dielectric constants of AlO_*x*_ and fibronectin were taken from refs 40 and 41. The surrounding medium above the fibronectin layer was modeled as PBS (ε_p_ = 1.79). To simulate the condition of cell attachment, the top surface of the fibronectin-coated 3QW-enhanced plasmonic substrate was further covered with a membrane layer (ε_m_ = 2.13, thickness = 10 nm) and cytoplasm (ε_c_ = 1.85).^15^ A continuous TM-polarized plane-wave source with a wavelength of 488 nm was positioned 200 nm above the Al nanoparticle surface and incident normally onto the structure. Moreover, the unit grid size was set to 2 nm in the *x*-direction and 0.8 nm in the *z*-direction.

### Fluorescence Lifetime Measurement

The fibronectin-attached sapphire, 0QW-, and 3QW-enhanced plasmonic substrates were immersed in a 1 μM Atto 488 solution for 4 h. The substrates were rinsed three times with ethanol and dried with nitrogen gas. The fluorescence lifetimes of the fluorophores on these substrates were analyzed using a time-resolved fluorescence measurement system (MicroTime 200, PicoQuant) based on the time-correlated single-photon counting technique. The samples were excited with light having a wavelength of 485 nm.

### Fluorescence Imaging System

The blinking fluorescence images of the HM-JF_526_ fluorophores were acquired using a custom-built fluorescence microscope, as illustrated in Figure S1. The excitation light was supplied by a solid-state laser with a wavelength of 473 nm. Excitation light was expanded with a beam expander to increase its diameter, and its illumination on the sample was controlled by a shutter. The excitation power densities for single fluorophore blinking fluorescence measurement and cell imaging were 156 W/cm^2^, respectively. After reflection by a dichroic mirror, excitation light was illuminated onto the sample through an objective lens (100× CFI Achromat, Nikon) to excite fluorescence using an epifluorescence excitation configuration. The emitted fluorescence was collected by the same objective, passed through the dichroic mirror (Semrock) and a bandpass filter (Semrock), and then imaged onto the EMCCD camera (iXon Ultra 897, Andor) through a lens to obtain the fluorescence images. A three-dimensional motorized stage was used to control the illumination area of the sample. The effective pixel size of the images was 106 nm/pixel, and the diameter of the field of view of the fluorescence imaging system was approximately 27 μm. A total of 8,000 frames of blinking fluorescence images were continuously captured with an exposure time of 50 ms. Single-molecule localization analysis was performed using the ThunderSTORM program to reconstruct the SMLM image.

### Statistical Analysis

Data in this manuscript were presented as mean ± standard deviation. Statistical evaluation adopted an unpaired two-tailed Student’s *t* test, and the significance levels were denoted as *P < 0.05, **P < 0.01, ***P < 0.001, and ****P < 0.0001.

## Results and Discussion

### Fluorescence Enhancement by Quantum Well-Enhanced Plasmonic Substrates

To investigate whether multi-QW-enhanced plasmonic substrates possess resonant charge transfer for fluorescence enhancement, we fabricated 0QW- and 3QW-enhanced plasmonic substrates, with their structural configurations shown in Figure 1a. The 3QW-enhanced plasmonic substrate is a multilayer structure that includes a sapphire substrate, a GaN nucleation layer, an n-GaN base layer, three repeated n-GaN barrier/InGaN quantum well layers, a GaN cap layer, and Al nanoparticles. The thickness of each layer in the 3QW-enhanced plasmonic substrate is shown in Table S1. The 0QW-enhanced plasmonic substrate does not include the three repeated n-GaN barrier/InGaN QW layers, providing a comparison to assess the influence of QWs on the enhancement and concentration of the surface charge density. Figure 1b presents scanning electron microscope (SEM) images of the 3QW-enhanced plasmonic substrates, revealing that the substrate surface exhibits nanoparticle-like Al structures. Furthermore, a stable AlO_*x*_ thin layer was rapidly generated on the surface of the Al nanospheres during the thermal annealing process, thereby preserving the underneath Al metal and facilitating the biocompatibility of the substrate.^42^

**Figure 1:**
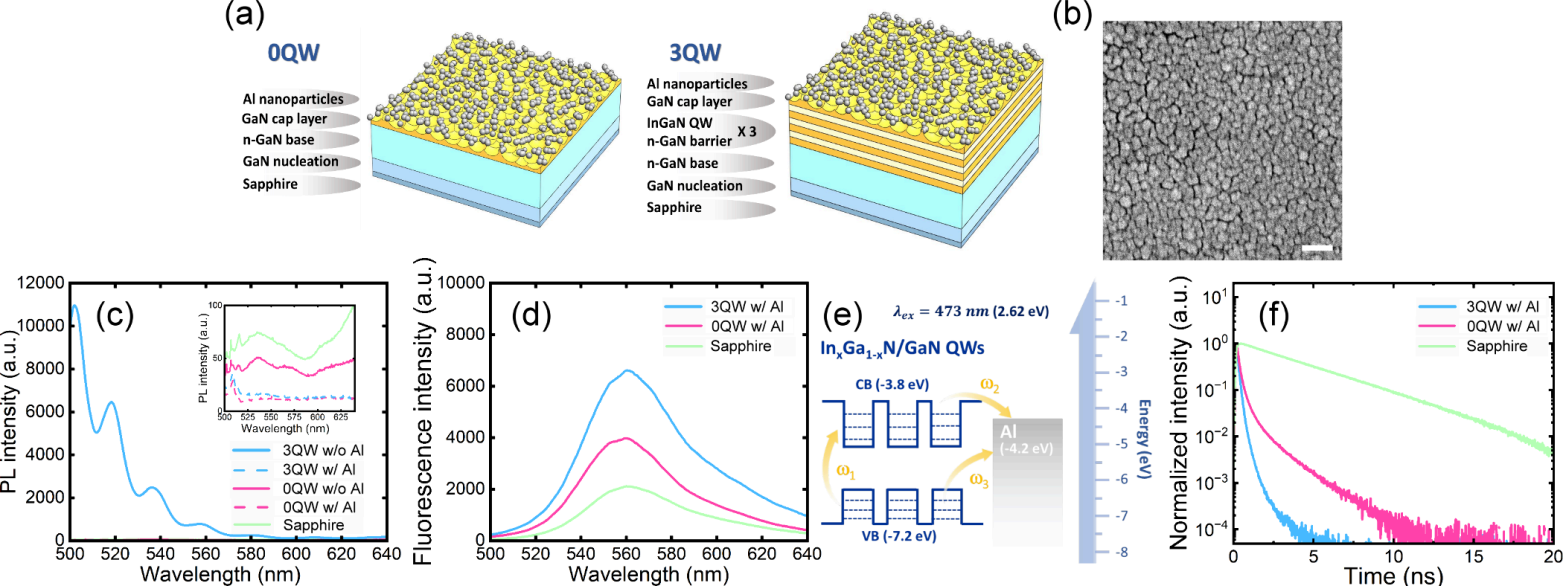
(a) Layer configuration of 0QW- and 3QW-enhanced plasmonic substrates. (b) SEM image to reveal the morphology of Al nanoparticles on 3QW-enhanced plasmonic substrates. Scale bar: 200 nm. (c) PL spectra of the sapphire, 0QW-, and 3QW-enhanced plasmonic substrates in the absence and presence of decorated Al nanoparticles for comparison. (d) Fluorescence spectra of R6G molecules on sapphire, substrates, 0QW-, and 3QW-enhanced plasmonic substrates. (e) Band alignment of the Al nanoparticles and the three repeated InGaN QWs in the 3QW-enhanced plasmonic substrate with respect to the vacuum level. The dashed lines within the QWs indicate discrete energy levels that bring additional charge-transfer channels (ω_1_, ω_2_, and ω_3_) for enhancing localized surface plasmon resonance. (f) Fluorescence lifetime decay curves of Atto 488 fluorophores on the three substrates.

We measured the photoluminescence (PL) spectra of sapphire, 0QW-, and 3QW-enhanced plasmonic substrates to assess the influence of background signals from these substrates on fluorescence signal measurements. Figure 1c shows the PL spectra of sapphire, 0QW-, and 3QW-enhanced plasmonic substrates with and without Al nanoparticle coating under 488 nm excitation. The PL signals from sapphire and 0QW substrate without Al nanoparticle coating exhibited very weak intensity. However, the 3QW substrate without Al coating exhibited notable PL spectral signals, with peak positions around 502 nm, covering a spectral range of approximately 500–580 nm. This notable PL signal is the result of the recombination processes related to InGaN QWs.^19,32,33^ The multiple peaks observed in the PL spectrum of the 3QW substrate without Al coating are likely attributed to slight variations in the thickness or indium composition among the individual quantum wells during the MOCVD growth process.^43,44^ However, when Al nanoparticle coatings were applied to the surfaces of the 0QW and 3QW substrates, the PL signals were markedly suppressed, as illustrated in the inset of Figure 1c. According to our previous study,^33^ the transmission electron microscopy (TEM) image and energy-dispersive spectroscopy mapping results indicate that the thickness of the Al nanoparticle layer is approximately 25–45 nm. This suppression mainly arises from the thicker Al nanoparticle layer, which blocks the direct optical excitation of the underlying InGaN QWs and thus prevents PL emission. However, TEM imaging reveals that the Al nanoparticle layer does not fully cover the 3QW substrate; instead, it contains thinned or cracked regions that may allow a small fraction of excitation light to penetrate.^33^ Despite this partial penetration, no detectable PL signal was observed from the 0QW and 3QW-enhanced plasmonic substrates. The PL generated by the small amount of transmitted excitation light may be quenched by the charge transfer effect in this Al nanoparticles/InGaN QWs structure, resulting in the absence of observable PL. This reduction in PL background greatly reduces its interference with single-molecule fluorescence detection. Additionally, the previous study measured the reflection spectrum of the 3QW-enhanced plasmonic substrate (i.e., the QW505 substrate) to investigate the coupling effect between the InGaN QWs and the localized surface plasmon resonance of Al nanoparticles, by varying the well width and indium composition of the InGaN QWs.^33^ A distinct reflection dip at 665 nm and a reduction in the Fabry–Perot oscillation strength in the 500–600 nm range were observed, which can be attributed to modifications of the Fabry–Perot interference by the wider QWs, as well as to the plasmonic coupling between the InGaN QWs and the localized surface plasmon resonance of the Al nanoparticles. The 3QW-enhanced plasmonic substrate exhibits such a broad resonance range due to the varied sizes of the Al nanoparticles and the discrete energy levels of the QWs.

For preventing fluorescence quenching, a layer of fibronectin proteins was attached to the sapphire, 0QW-, and 3QW-enhanced plasmonic substrates to create a spacing between the fluorophore and the Al nanoparticle. This fibronectin layer can also promote cell adhesion.^15^ After depositing 1 μM R6G molecules onto the surfaces of sapphire, 0QW-, and 3QW-enhanced plasmonic substrates, we measured the fluorescence spectra of R6G. The samples were excited with a 488 nm laser at a power of 3.6 mW to compare the fluorescence enhancement across the different substrates. As illustrated in Figure 1d, R6G showed the strongest fluorescence signal on the 3QW-enhanced plasmonic substrate, achieving a peak intensity enhancement of approximately 166% compared to the 0QW-enhanced plasmonic substrate. In addition to the localized surface plasmon enhancement effect caused by Al nanoparticles,^45,46^ the Al nanoparticles/InGaN QW structure of the 3QW-enhanced plasmonic substrate can facilitate multiple channels of charge transfer resonance.^32,33^ Because the incorporation of three repeated InGaN QWs beneath the substrate surface enhances the piezoelectric polarization, the internal electric field and band tilting within the QWs are strengthened to promote greater electron accumulation near the surface.^35^ Furthermore, upon optical excitation, a portion of the high-density electrons near the surface can transfer to the Al nanoparticle surface either by overcoming the Schottky barrier or via tunneling.^35,47^ The additional resonant charge transfer mechanism can be elucidated using the band diagram of the Al nanoparticles and three repeated InGaN QWs in the 3QW-enhanced plasmonic substrate, as shown in Figure 1e. The excitons in the valence band of the InGaN QWs can be excited to the conduction band (ω_1_), and part of the excitons can be subsequently relaxed to the Al surface (ω_2_). Other excitons in the valence band are also possible to be excited directly to the Al surface (ω_3_). These additional resonant charge transfers enhance the localized surface plasmon resonance effect, increasing the fluorescence signals of the R6G molecules. InGaN QWs also demonstrate the ability to enhance surface charge concentration, further promoting these resonance effects of charge transfer.^35^ Additionally, the high-density confined surface charges in the InGaN QW substrate can interact with the localized surface plasmons of Al nanoparticles.^34–36^ This plasmonic interaction generates an additional plasmonic enhancement, which not only enhances the signal, but also increases the density of localized enhancement regions. Thus, these combined effects contribute significantly to the overall enhancement of the fluorescence signal of R6G molecules on the 3QW-enhanced plasmonic substrate. Compared with conventional plasmon-enhanced fluorescence, which relies solely on the localized surface plasmon resonance of metal nanoparticles or nanostructures to amplify fluorescence signals, the proposed 3QW-enhanced plasmonic substrate provides multiple enhancement mechanisms. Furthermore, unlike surface plasmon polariton-enhanced or surface plasmon-coupled emission substrates that require specific excitation angles to achieve fluorescence enhancement,^15,22^ the 3QW-enhanced plasmonic substrate alleviates such angular constraints, thereby simplifying the optical excitation requirement in the imaging system. Without the multiple enhancement effects of the Al nanoparticles and the InGaN QWs, the fluorescence signal of the R6G molecules on the sapphire substrate is noticeably lower than that on the 3QW-enhanced plasmonic substrate.

Furthermore, the sapphire, 0QW-, and 3QW-enhanced plasmonic substrates were coated with a layer of Atto 488 fluorophores to investigate the variation of fluorescence lifetime. Figure 1f presents the fluorescence lifetime decay curves of Atto 488 fluorophores on sapphire, 0QW-, and 3QW-enhanced plasmonic substrates. The average fluorescence lifetimes of Atto 488 on the sapphire, 0QW-, and 3QW-enhanced plasmonic substrates were approximately 4.00, 0.77, and 0.25 ns, respectively. The sapphire substrate exhibits the typical fluorescence lifetime of Atto 488. In contrast, the 3QW-enhanced plasmonic substrate shows the shortest lifetime due to the multiple enhancement mechanisms of localized surface plasmon resonance and InGaN QWs. Compared with the 3QW-enhanced plasmonic substrate, the 0QW-enhanced plasmonic substrate shows a longer fluorescence lifetime, as it provides only Al nanoparticle-induced localized surface plasmon resonance enhancement without the additional enhancement effects of QWs.

The electric field distribution on the 3QW-enhanced plasmonic substrate was simulated using the finite-difference time-domain method. In the simulation, the top surface of the Al nanoparticles on the 3QW-enhanced plasmonic substrate was covered by an approximately 5 nm thick AlO_*x*_ layer, consistent with previous reports.^33^ A 4 nm fibronectin layer was also included on the top surface of the AlO_*x*_ layer, and the open-edge structure of the Al nanoparticles was modeled.^33,40^ Under normally incident TM-polarized light at a wavelength of 488 nm, the distribution of the simulated electric field of the substrate in PBS solution (Figure S2a) shows a slight enhancement on the nanoparticle surface and the strongest fields concentrated at the nanoparticle edges. The range of electric field enhancement, defined by the distance over which the field intensity decays to 1/e of its maximum value, was estimated to be approximately 36 nm. Moreover, as shown in Figure S2a–c, both the magnitude and range of the electric field enhancement do not exhibit any significant differences when the AlO_*x*_ and fibronectin layers were included, compared to the case without these layers. In addition, the presence of adherent cells on the substrate surface slightly increases the electric field enhancement, as shown in Figure S2d. During imaging of transmembrane proteins on the basal cell membrane, the axial position of the transmembrane proteins and the size of the primary and secondary antibodies increase the distance between the fluorophores and the substrate.^15,48^ Assuming a maximum fluorophore-substrate distance of approximately 60 nm, the electric field enhancement effect is attenuated at this position. To improve enhancement efficiency, alternative approaches may adopt nanobody-based or small-molecule labeling strategies to minimize this distance. Because the enhancement region is confined near the substrate surface, the 3QW-enhanced plasmonic substrate primarily enhances fluorescence signals from regions in close proximity to the surface.

Furthermore, we performed a comparative analysis of the absorption spectra for 0QW- and 3QW-enhanced plasmonic substrates, as shown in Figure S3. The absorption of the 3QW-enhanced plasmonic substrate does not exhibit a significant increase compared to that of the 0QW-enhanced plasmonic substrate. This result indicates that the enhanced blinking fluorescence on the 3QW-enhanced plasmonic substrates is not attributable to a change in the absorption cross-section.

### Fluorescence Blinking Measurement of a Single Spontaneously Blinking Fluorophore

To perform SMLM imaging, we evaluated the enhancement effect on the blinking fluorescence of a single spontaneously blinking fluorophore (HM-JF_526_)^12^ on a 3QW-enhanced plasmonic substrate. Figure 2a illustrates the time traces of fluorescence intensities of a single HM-JF_526_ under 488 nm excitation on sapphire, 0QW, and 3QW-enhanced plasmonic substrates. HM-JF_526_ exhibited significantly higher blinking fluorescence on the 3QW-enhanced plasmonic substrate compared to the sapphire and 0QW-enhanced plasmonic substrates. Consequently, we define the intensity threshold as *S*_T_ = *S*_AVG_ + 3*S*_STD_, where *S*_AVG_ and *S*_STD_ represent the average and standard deviation of the background signal, respectively.^15^ Using this threshold, the fluorescence-on signals in the fluorophore intensity trajectories were extracted to obtain the fluorescence intensity distributions illustrated in Figure 2b. The average intensities of fluorescence-on signals for HM-JF_526_ on sapphire, 0QW, and 3QW-enhanced plasmonic substrates were approximately 524, 938, and 1755 au, respectively. HM-JF_526_ on the 3QW-enhanced plasmonic substrate exhibits a higher fluorescence intensity than on the 0QW-enhanced plasmonic substrate. The 3QW-enhanced plasmonic substrate provides multiple enhancements of the local surface plasmon resonance effect, including additional channels for charge transfer resonance, a high concentration of surface charges, local surface plasmon resonance from Al nanoparticles, and surface plasmon coupling between Al nanoparticles and InGaN QWs.^30,31,33–36^ The enhancement of the local surface plasmon resonance effect on the substrates not only increases the local electromagnetic field and the fluorophore’s absorption and emission cross sections, but more importantly, induces optical resonance coupling with the fluorophores. This coupling enhances the excitation rate and emission coupling efficiency, and modifies the quantum yield and fluorescence lifetime, thereby increasing the blinking fluorescence signals of the spontaneously blinking fluorophores.^15,20,21,24^ Consequently, we can achieve a 335% enhancement of the HM-JF_526_ blinking fluorescence signal on the 3QW-enhanced plasmonic substrate, compared to the signal on the sapphire substrate. Furthermore, the number of blinking events of HM-JF_526_ is also significantly increased on the 3QW-enhanced plasmonic substrate compared to the sapphire and 0QW-enhanced plasmonic substrates, as shown in Figure 2c. Therefore, 3QW-enhanced plasmonic substrates improve the detection of photons emitted by individual HM-JF_526_ fluorophores and significantly increase the number of detectable blinking events, resulting in a greater number of localization events during SMLM imaging. A sufficient localization density in SMLM images is essential for obtaining high-resolution imaging.^4^

**Figure 2:**
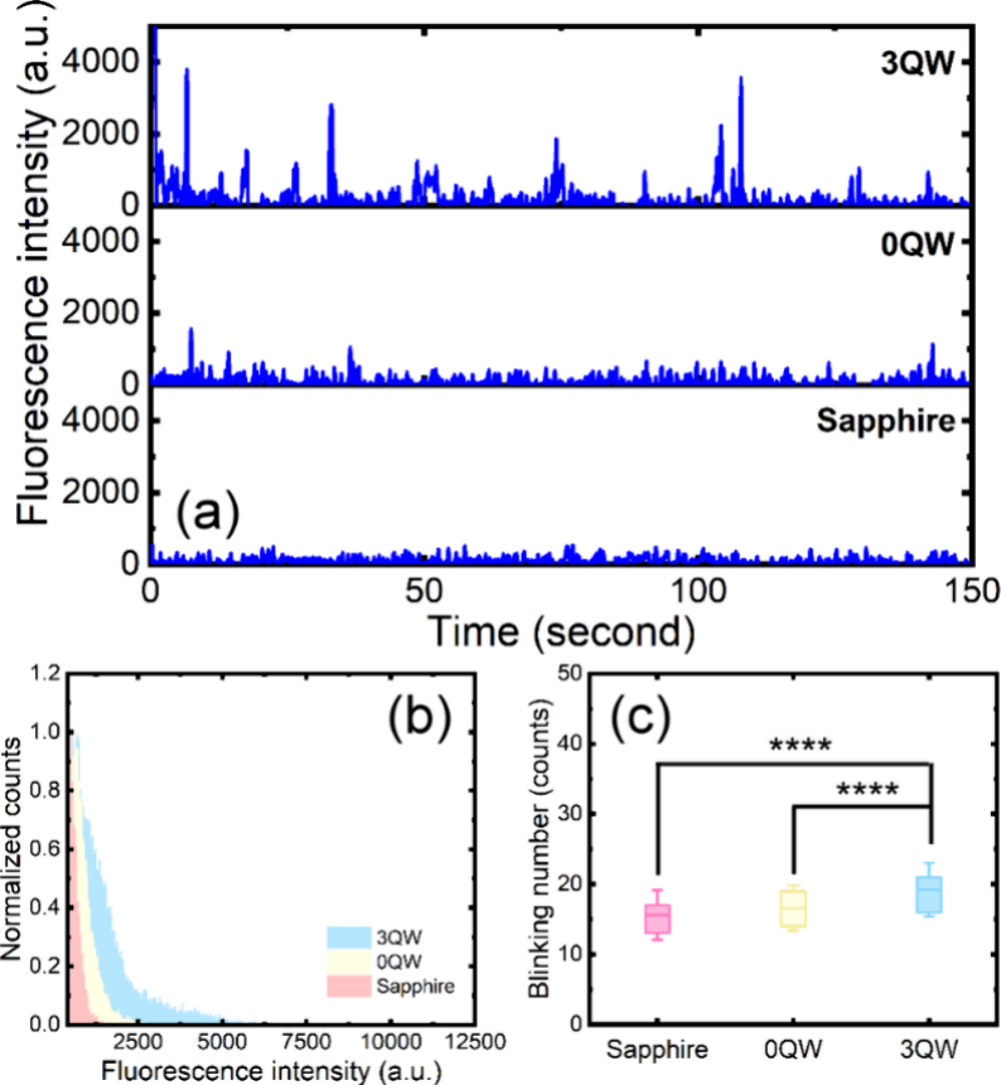
(a) Time trace of fluorescence signals from a single HM-JF_526_ fluorophore on sapphire, 0QW-, and 3QW-enhanced plasmonic substrates to compare the enhancement of blinking fluorescence. (b) Normalized histograms of the fluorescence intensity of an HM-JF_526_ fluorophore in the fluorescence-on state on sapphire, 0QW-, and 3QW-enhanced plasmonic substrates. (c) Fluorescence blinking events of an HM-JF_526_ fluorophore on the three substrates. The box denotes the first and third quartiles, the line denotes the mean value, and the whisker denotes the standard deviation of the distribution. The mean number of blinking events on each substrate was calculated from more than 40 time traces of fluorescence signals from the HM-JF_526_ fluorophore.

### SMLM Imaging of EGFRs in A549 Lung Cancer Cells

EGFR, also called ERBB1/HER1, is a member of the ErbB family of receptor tyrosine kinases. When EGFR binds to its ligand, it undergoes homodimerization or heterodimerization, which leads to autophosphorylation of intracellular tyrosine residues. This process activates downstream signaling pathways that promote cell survival, proliferation, and migration.^49–51^ However, overexpression of EGFR signaling is highly associated with cancer cell survival, proliferation, metastasis, tumor angiogenesis, and drug resistance. We conducted SMLM imaging of HM-JF_526_-labeled EGFRs in A549 lung cancer cells on glass coverslip, 0QW-, and 3QW-enhanced plasmonic substrates to evaluate cell imaging performance. Figure 3a–c show the SMLM images of EGFRs in A549 lung cancer cells on the three substrates, using an excitation power density of 156 W/cm^2^. At low excitation power density, the glass coverslip showed a sparse distribution of EGFRs, mainly attributed to a few high-brightness localization events. In contrast, both the 0QW and 3QW-enhanced plasmonic substrates exhibited a higher number of EGFR localization events. Notably, the 3QW-enhanced plasmonic substrate showed a denser distribution of EGFRs, along with typical clustering patterns.

**Figure 3:**
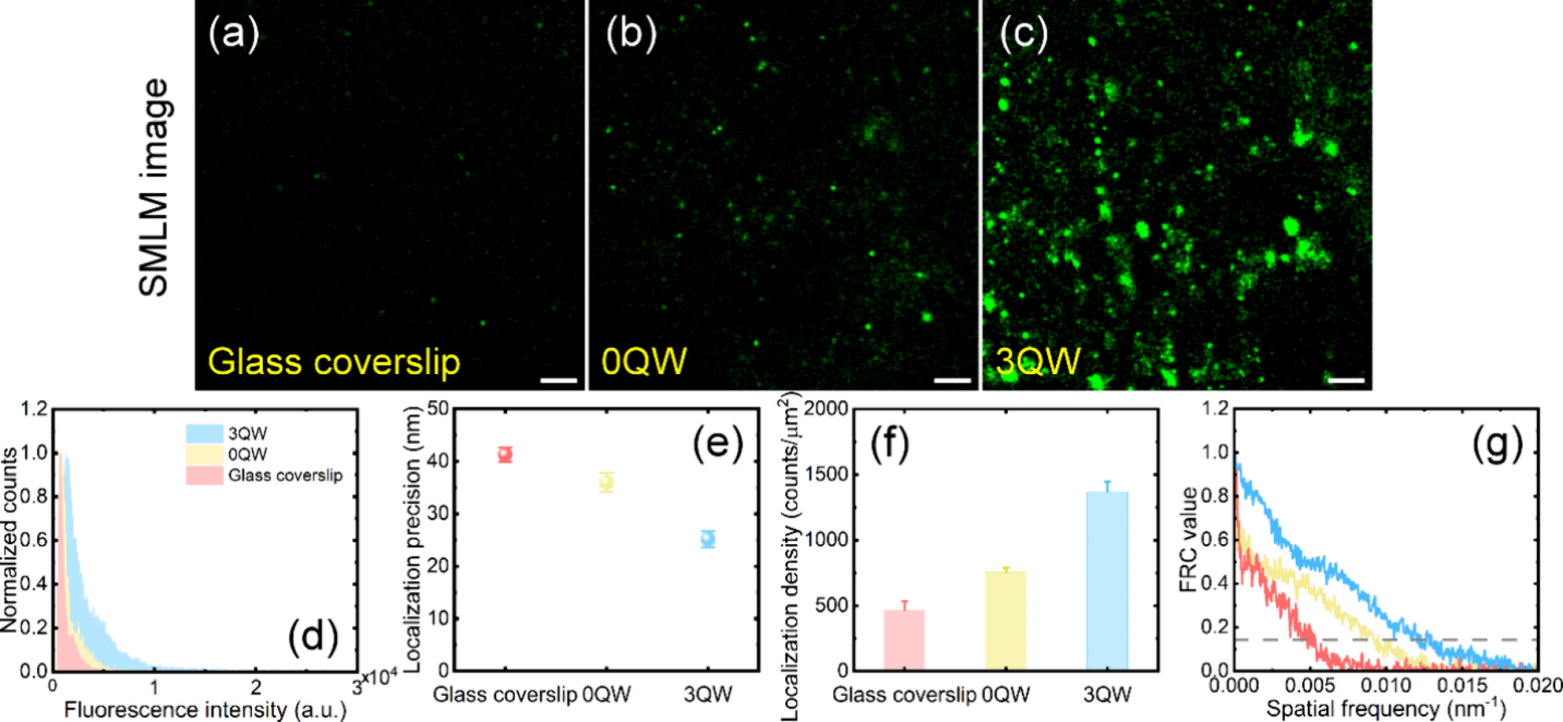
(a–c) SMLM images of the HM-JF_526_-labeled EGFRs in A549 lung cancer cells on glass coverslip, 0QW-, and 3QW-enhanced plasmonic substrates. Scale bars: 1 μm. (d) Normalized histograms of the fluorescence intensity distribution in each localization event of HM-JF_526_-labeled EGFRs on the three substrates in panels (a–c). (e, f) Results of comparison of the localization precision (e) and density (f) of HM-JF_526_-labeled EGFRs on the three substrates. (g) FRC curves for SMLM images of HM-JF_526_-labeled EGFRs in A549 lung cancer cells shown in panels (a–c).

The fluorescence intensities of the localization events on the three substrates were statistically analyzed to estimate the enhancement of fluorescence in the SMLM images, as illustrated in Figure 3d. The average fluorescence intensities of localization events were approximately 1981, 3049, and 6463 au for the glass coverslip, 0QW-, and 3QW-enhanced plasmonic substrates, respectively. Consequently, the localization precision of the SMLM images was calculated for the three substrates, as illustrated in Figure 3e. The 3QW-enhanced plasmonic substrates significantly improved the localization precision of the HM-JF_526_-labeled EGFR in SMLM images to approximately 25 nm. Moreover, the localization density was calculated using the distribution of localization events in the SMLM images, with the results presented in Figure 3f. The localization density on the 3QW-enhanced plasmonic substrates was significantly higher than that on the glass coverslip, mainly because more blinking events were detectable during SMLM imaging, as shown in Figure 2c. Although there was a slight increase in localization density on the 0QW-enhanced plasmonic substrates, the overall increase remained limited due to the lack of contributions from the QWs. Additionally, the discrepancy between the significant increase in localization density shown in Figure 3f and the relatively small increase in blinking events in Figure 2c arises from the different thresholding methods. The identification of fluorescence-on signals in the fluorophore intensity trajectory of a HM-JF_526_ fluorophore is based on a simple intensity threshold. However, the SMLM localization algorithm uses more complex criteria to select appropriate blinking events for the final reconstructed image. The threshold criteria are not only based on the intensity of the blinking event but also on the quality of the fitted point spread function (PSF).^52^ The weak fluorescence signal results in a low signal-to-noise ratio (SNR), which causes the PSF pattern to appear incomplete and consequently be rejected by the algorithm as noise. The blinking fluorescence signals of HM-JF_526_ fluorophores on the 3QW-enhanced plasmonic substrate exhibit a markedly higher SNR compared with those observed on the other two substrates, as shown in Figures 2a and 3d. Therefore, a high SNR fluorescence signal yields well-defined PSFs, ensuring accurate fitting and retention of localization information, which in turn markedly enhances localization density. Furthermore, according to the Nyquist–Shannon criterion, a higher localization density is critical to improve the Nyquist resolution of SMLM images.^53^ Nyquist resolution can be calculated using the formula $\sigma_{\mathrm{Nyquist}}=2/\sqrt{\rho }$ , where ρ represents the localization density of the SMLM image. The Nyquist resolutions for the glass coverslip, 0QW-, and 3QW-enhanced plasmonic substrates were approximately 93, 73, and 54 nm, respectively. However, the image resolution in SMLM should consider both localization precision and density. Thus, we estimated the SMLM image resolution using the Fourier ring correlation (FRC) method.^54^ The FRC resolution is determined by randomly dividing a reconstructed image data set into two subsets and calculating the spatial frequency at which the FRC value falls to 1/7. This spatial frequency is defined as the FRC resolution of the SMLM image, as illustrated in Figure 3g. The FRC resolution of the SMLM image obtained from the 3QW-enhanced plasmonic substrates reached 75 ± 1 nm, showing a significant improvement over the glass coverslip and 0QW-enhanced plasmonic substrates.

However, the size range of EGFR clusters in the plasma membrane of lung cancer cells spans from 70 to 660 nm.^55^ This large variation makes it difficult to define a standard cluster size from the EGFR imaging data for validating the image resolution. Therefore, to evaluate the SMLM imaging resolution on the 3QW-enhanced plasmonic substrate, we further performed SMLM imaging of microtubules, which are cellular structures with a well-defined width. The outer diameter of a single microtubule is approximately 25 nm.^56^ Figure S4 shows the SMLM image of HM-JF_526_-labeled microtubules on the 3QW-enhanced plasmonic substrates. The cross-section intensity profile of a single microtubule was fitted with a Gaussian function, and the full width at half-maximum of the fitted curve was regarded as the width of the microtubule. The average width of the microtubule obtained from the SMLM images was 65 ± 1 nm (*n* = 25). This measured width is larger than the actual value, primarily due to the size effect of the primary and secondary antibodies in the immunostaining.^10^ The measured width is consistent with the values reported in previous studies.^5,10^

Therefore, the 3QW-enhanced plasmonic substrates improve the fluorescence signals and blinking events of HM-JF_526_ fluorophores, resulting in enhanced resolution in SMLM imaging. Furthermore, the enhanced fluorescence blinking of the HM-JF_526_ fluorophore on the 3QW-enhanced plasmonic substrates suggests that the acceptable image resolution of SMLM can be achieved with a lower excitation power density compared to SMLM imaging performed on a glass coverslip. Relying solely on intense local electromagnetic field enhancement to increase fluorescence signals can still lead to the photodamage of the sample. However, the localized surface plasmon resonance on 3QW-enhanced plasmonic substrates increases fluorescence intensity not only by enhancing the local electromagnetic field but also by modulating the photophysical properties of the fluorophores via the Purcell effect. Specifically, the localized surface plasmon resonance on 3QW-enhanced plasmonic substrates enhances the local density of optical states, which creates an optical resonance coupling with the fluorophores.^15,20,21,24,57^ This coupling accelerates the radiative decay rate, thereby modifying the quantum yield and fluorescence lifetime, which subsequently increases the blinking fluorescence signal. Consequently, the fluorescence enhancement provided by the 3QW-enhanced plasmonic substrates is not solely attributable to local electromagnetic field enhancement. Compared with the approach that relies exclusively on high excitation power density to obtain sufficient fluorescence signals, the 3QW-enhanced plasmonic substrates achieve comparable fluorescence signals and acceptable SMLM resolution at a significantly lower excitation power density. As a result, SMLM imaging performed on the 3QW-enhanced plasmonic substrates with the lower excitation power density reduces the probability of photodamage to the sample.

### EGFR Signaling Inhibition of A549 Lung Cancer Cells with Tyrosine Kinase Inhibitor Treatment

TKIs can bind to the tyrosine kinase domain of EGFR, preventing adenosine triphosphate from binding and thus inhibiting EGFR phosphorylation.^58,59^ This process effectively blocks the activation of its kinase. Inhibition of TKI-induced kinases disrupts downstream signaling pathways of EGFR, including the JAK/STAT, PI3K/AKT, and RAS/RAF/MEK/ERK pathways. This disruption leads to reduced survival and proliferation of tumor cells. Gefitinib is a first-generation oral active EGFR-TKI that has been approved in numerous countries as a targeted therapy for patients with advanced nonsmall cell lung cancer.^60,61^ Gefitinib has exhibited good clinical tolerability and antitumor efficacy. We used SMLM imaging on 3QW-enhanced plasmonic substrates to examine the inhibition level of EGFR tyrosine kinase activation in A549 lung cancer cells treated with Gefitinib. Figure 4a–c display SMLM images of phosphorylated EGFR (pEGFR) in A549 lung cancer cells cultured at different concentrations of Gefitinib, captured at an excitation power density of 156 W/cm^2^. In the absence of Gefitinib inhibition (Figure 4a), a significant amount of pEGFR expression exhibiting typical clustering patterns was observed in A549 lung cancer cells on 3QW-enhanced plasmonic substrates. When 5 μM and 10 μM of Gefitinib were present, the number of pEGFR in A549 lung cancer cells decreased significantly. Additionally, the density of pEGFR in the SMLM images was calculated, as illustrated in Figure 4d. The densities of pEGFR at 0, 5, and 10 μM Gefitinib were measured at approximately 1327, 558, and 164 counts/μm^2^, respectively. The density of pEGFR exhibited a substantial reduction when treated with high concentrations of Gefitinib, demonstrating a concentration-dependent inhibitory trend. The 3QW-enhanced plasmonic substrates provide multiple enhancements of the local surface plasmon resonance effect. The enhancement of the local surface plasmon resonance effect on the 3QW-enhanced plasmonic substrates not only increases the excitation rate of fluorophores through local electromagnetic field enhancement but also enhances the radiative decay rate by increasing the local density of optical states, thereby modulating the quantum yield and fluorescence lifetime of the fluorophores and leading to increased blinking fluorescence signals.^15,20,21,24,57^ Furthermore, an increased radiative decay rate further suppresses photobleaching and improves fluorophore photostability, enabling the detection of more blinking events and thereby increasing the localization events in SMLM imaging. The 3QW-enhanced substrates significantly improve SMLM image resolution by simultaneously enhancing the blinking intensity and the number of blinking events of spontaneously blinking fluorophores during SMLM imaging. Therefore, compared with SMLM imaging on glass coverslips, SMLM images of pEGFRs in A549 lung cancer cells on 3QW-enhanced plasmonic substrates can be obtained even at low excitation power density, enabling a quantitative analysis of the extent of EGFR tyrosine kinase inhibition during TKI treatment. However, the localized surface plasmon resonance enhancement induced by the randomly distributed Al nanoparticles and the charge-transfer resonances of the QWs on the 3QW-enhanced plasmonic substrate are not uniformly distributed. Figure S5 shows an SMLM image of a uniformly coated HM-JF_526_ layer on a fibronectin-attached 3QW-enhanced plasmonic substrate. This nonuniform enhancement can influence the distribution of target molecules in fluorescence imaging. Therefore, the 3QW-enhanced plasmonic substrate should primarily be regarded as a platform for fluorescence signal enhancement.

**Figure 4:**
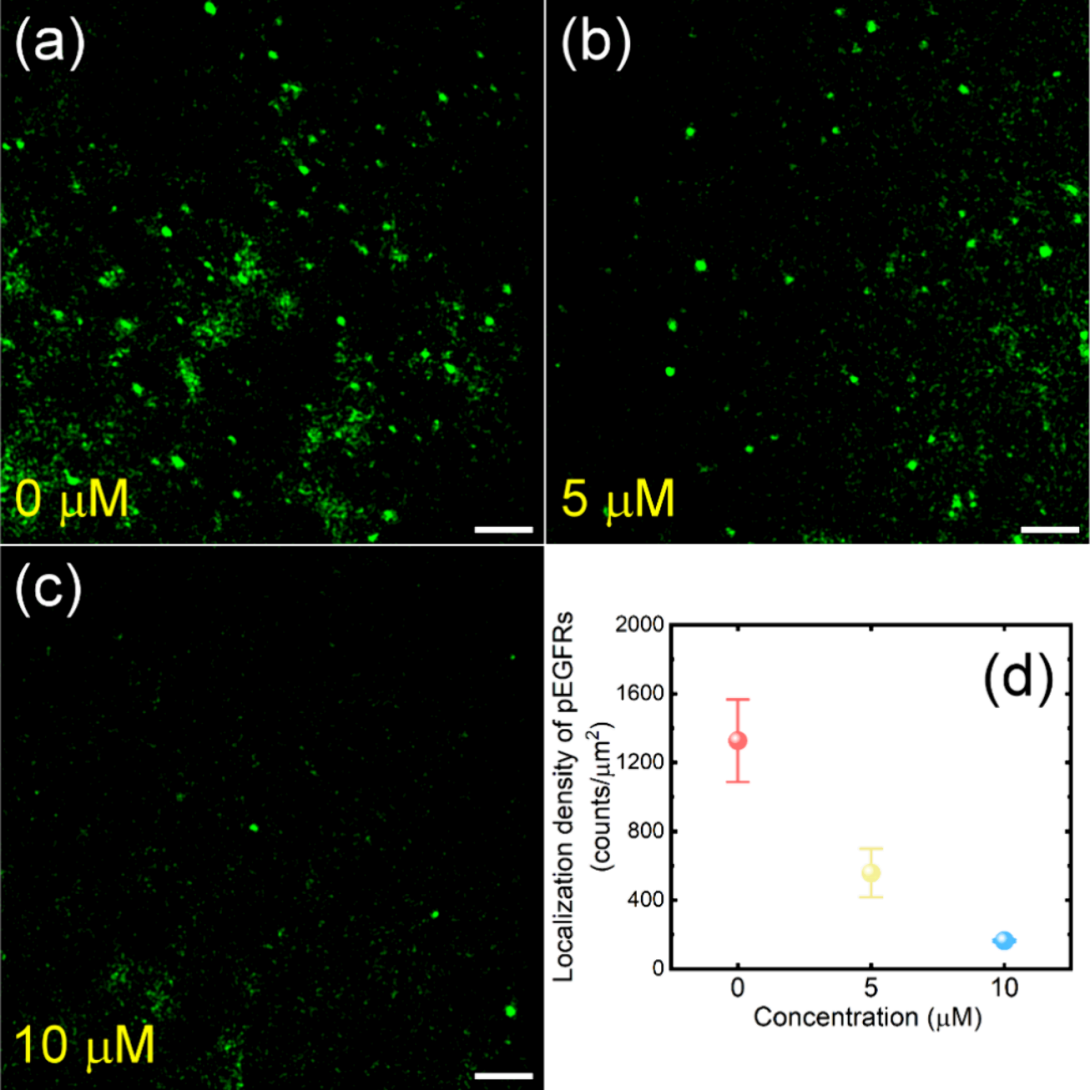
(a–c) SMLM images of the HM-JF_526_-labeled pEGFRs in A549 lung cancer cells after incubation with different concentrations of Gefitinib for 40 h. Scale bars: 1 μm. (d) Quantitative analysis of the localization densities of pEGFRs in panels (a–c) to evaluate the inhibition of the EGFR tyrosine kinase.

## Conclusions

In conclusion, we fabricated a wafer-scale three-repeat InGaN QW structure on sapphire substrates using MOCVD. The three-repeat InGaN QWs allow for a high concentration of charges to accumulate at the substrate surface and provide tunable discrete energy levels, which serve as an additional source for charge transfer. A layer of Al nanoparticles deposited on the surface of the InGaN QW substrate induces a localized surface plasmon resonance effect. Moreover, the Al nanoparticles/InGaN QWs structure also creates additional channels for charge transfer resonance to enhance the local surface plasmon resonance on the surface of Al nanoparticles. Consequently, a single spontaneously blinking fluorophore exhibited significantly increased blinking fluorescence and events on the 3QW-enhanced plasmonic substrate. This 3QW-enhanced plasmonic substrate also improved localization precision and density in SMLM imaging, thus enhancing image resolution to visualize EGFR distribution in A549 lung cancer cells. Furthermore, the amount of pEGFR localization in A549 lung cancer cells on the 3QW-enhanced plasmonic substrate was quantified using SMLM imaging to examine inhibition of EGFR tyrosine kinase during TKI treatment. Therefore, the significant increases in blinking fluorescence signals and localization events also indicate the lower excitation power needed to achieve acceptable resolution in SMLM imaging, which will facilitate high-resolution imaging analysis.

## Supplementary Material



## Notes

Optical configuration of SMLM imaging and the MOCVD fabrication parameters for the 3QW-enhanced plasmonic substrates (PDF)
