# Supplementary material for: Quantum Well-Enhanced Plasmonic Substrate to Enhance Spontaneously Blinking Fluorescence for Single-Molecule Localization Microscopy
Source: Anal Chem. 2026 Mar 10;98(11):7999–8009. doi: 10.1021/acs.analchem.5c03721 (PMC13019428; doi:10.1021/acs.analchem.5c03721)
Supplement: Supplementary file 1 [file ac5c03721_si_001.pdf]

Supporting Information

Quantum Well-Enhanced Plasmonic Substrate to Enhance Spontaneously Blinking Fluorescence for Single-Molecule Localization Microscopy

Shang-En Hsieh[†], *Jian-Zong Lai*[†], *Kun-Yu Lai*[†,*], *Wan-Chen Huang*[‡,§], *and Fan-Ching Chien*[†,*]

[†]Department of Optics and Photonics, National Central University, Taoyuan 32001, Taiwan.

[‡]Single-Molecule Biology Core Lab, Institute of Cellular and Organismic Biology, Academia *Sinica, Taipei 115201, Taiwan.*

[§]Institute of Medical Device and Imaging, National Taiwan University, Taipei 10051, Taiwan.

E-mail: kylai@ncu.edu.tw and fcchien@dop.ncu.edu.tw

Table of Contents

Figure S1. The optical configuration of the SMLM imaging on the 3QW-enhanced plasmonic substrates........ S2

Table S1. The MOCVD fabrication parameters for the 3QW-enhanced plasmonic substrates........ S3

Figure S2. Simulated electric field distributions of the 3QW-enhanced plasmonic substrates with (a) AlO_x and fibronectin layers; (b) a fibronectin layer only; (c) an AlO_x layer only; and (d) AlO_x, fibronectin, and adhesion cell layers........ S4

Figure S3. The absorption spectra of 0QW- and 3QW-enhanced plasmonic substrates........ S5

Figure S4. The SMLM imaging of cell microtubules labeled with HM-JF_{526} fluorophores on the 3QW-enhanced plasmonic substrate........ S6

Figure S5. The SMLM image of a HM-JF_{526} fluorophore layer coated on a 3QW-enhanced plasmonic substrate........ S7

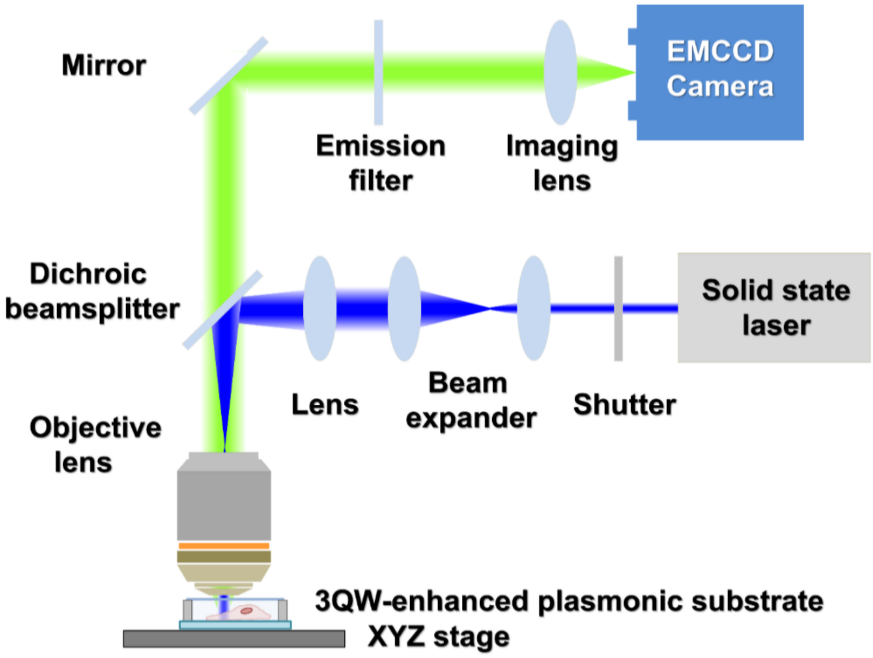

Figure S1. The Optical configuration of the SMLM imaging on the 3QW-enhanced plasmonic substrates.

Table S1. The MOCVD fabrication parameters for the 3QW-enhanced plasmonic substrates.

Layer material	Layer thickness (nm)	MOCVD temperature (°C)
GaN cap layer	1.6	775
InGaN QW layer	2	775
n-GaN barrier layer	3	775
InGaN QW layer	2	775
n-GaN barrier layer	3	775
InGaN QW layer	2	775
n-GaN barrier layer	3	775
n-GaN base layer	2000	1120
GaN nucleation layer	25	550
Sapphire substrate	-	-

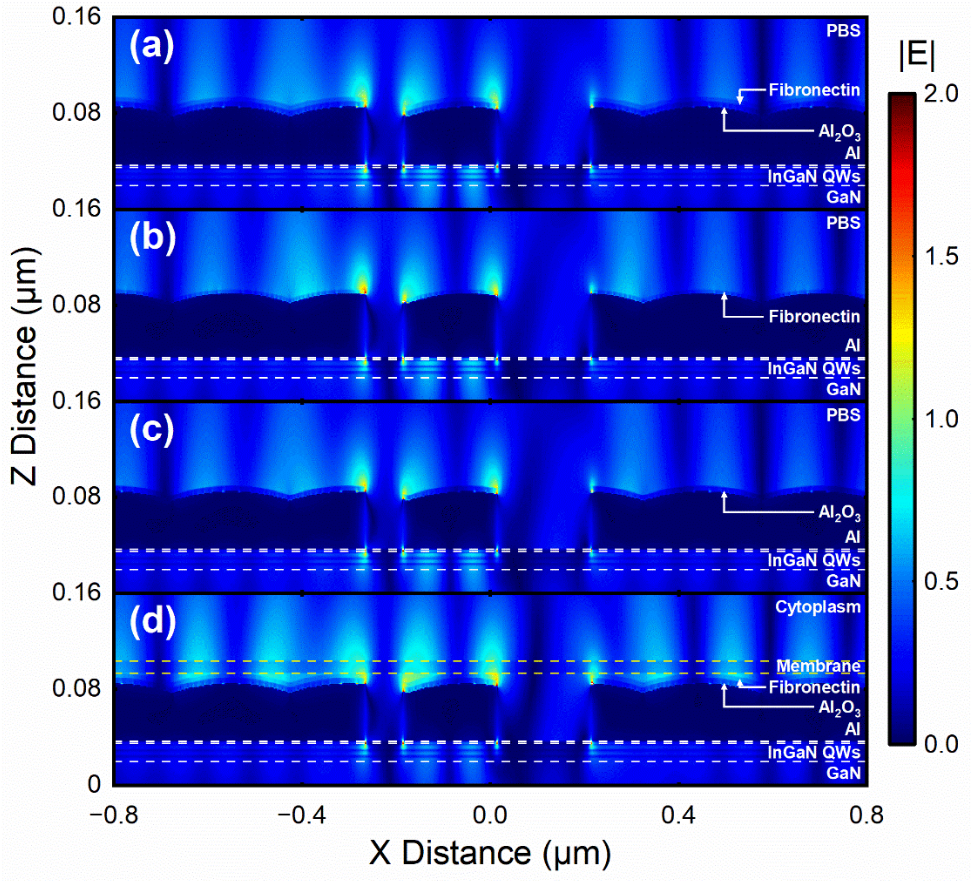

Figure S2. Simulated electric field distributions of the 3QW-enhanced plasmonic substrates with (a) AlO_x and fibronectin layers; (b) a fibronectin layer only; (c) an AlO_x layer only; and (d) AlO_x, fibronectin, and adhesion cell layers.

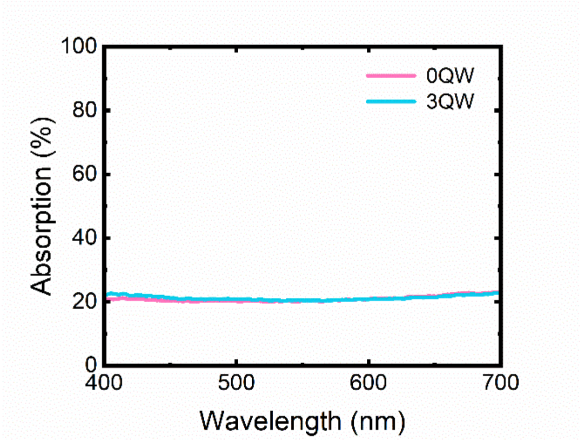

Figure S3. The absorption spectra of 0QW- and 3QW-enhanced plasmonic substrates.

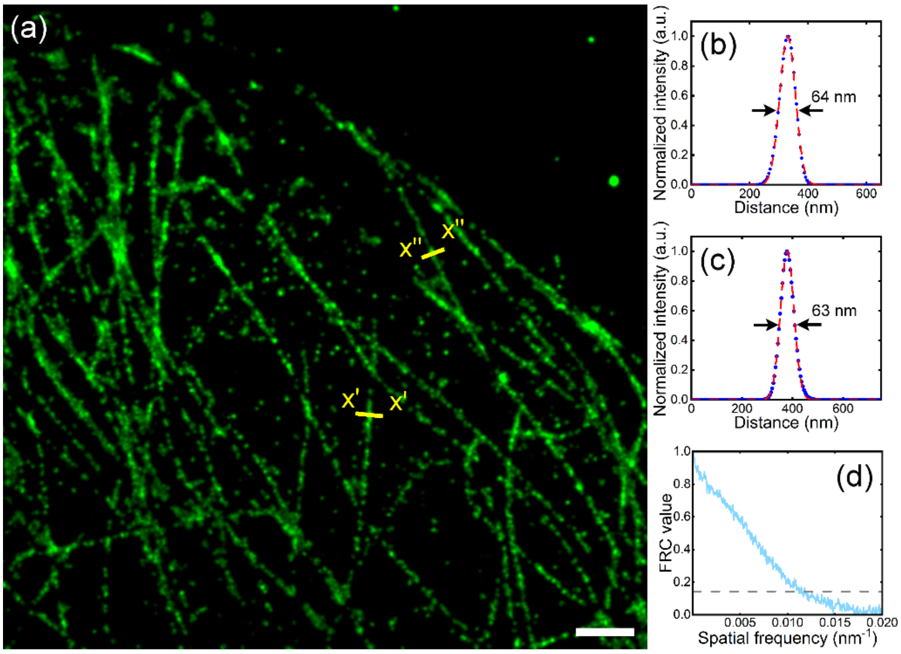

Figure S4. (a) The SMLM image of cell microtubules labeled with HM-JF526 fluorophores on the 3QW-enhanced plasmonic substrate. Scale bar: 1 μm. (b), (c) Cross-sections of a single microtubule along the yellow lines (x'-x' and x''-x'') in panel (a). The cross-section intensity profile was fitted by the Gaussian equation to determine its full width at half maximum. The blue dots and red dashed lines represent the raw data and fitting results, respectively. (d) FRC calculation for the SMLM image data in panel (a). The gray dashed line indicates the threshold at an FRC value of 1/7. The FRC resolution of the SMLM image data in panel (a) is approximately 65 ± 1 nm.

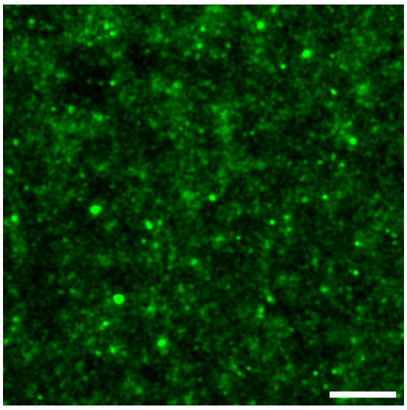

Figure S5. The SMLM image of a HM-JF_{526} fluorophore layer coated on a 3QW-enhanced plasmonic substrate. Scale bar: 1 μm.
